# Machine learning-optimized non-invasive brain stimulation and treatment response classification for major depression

**DOI:** 10.1186/s42234-024-00157-2

**Published:** 2024-10-30

**Authors:** Alejandro Albizu, Aprinda Indahlastari, Paulo Suen, Ziqian Huang, Jori L. Waner, Skylar E. Stolte, Ruogu Fang, Andre R. Brunoni, Adam J. Woods

**Affiliations:** 1 Center for Cognitive Aging and Memory, McKnight Brain Institute, University of Florida, Gainesville, USA; 2 Department of Neuroscience, College of Medicine, University of Florida, Gainesville, USA; 3 J. Crayton Pruitt Family Department of Biomedical Engineering, Herbert Wertheim College of Engineering, University of Florida, Gainesville, USA; 4 Department of Electrical and Computer Engineering, Herbert Wertheim College of Engineering, University of Florida, Gainesville, USA; 5 Department of Clinical and Health Psychology, College of Public Health and Health Professions, University of Florida, 1225 Center Drive, PO Box 100165, Gainesville, FL 32610-0165 USA; 6 Faculdade de Medicina da Universidade de São Paulo, São Paulo, Brasil

## Abstract

**Background/Objectives:**

Transcranial direct current stimulation (tDCS) is a non-invasive brain stimulation intervention that shows promise as a potential treatment for depression. However, the clinical efficacy of tDCS varies, possibly due to individual differences in head anatomy affecting tDCS dosage. While functional changes in brain activity are more commonly reported in major depressive disorder (MDD), some studies suggest that subtle macroscopic structural differences, such as cortical thickness or brain volume reductions, may occur in MDD and could influence tDCS electric field (E-field) distributions. Therefore, accounting for individual anatomical differences may provide a pathway to optimize functional gains in MDD by formulating personalized tDCS dosage.

**Methods:**

To address the dosing variability of tDCS, we examined a subsample of sixteen active-tDCS participants’ data from the larger ELECT clinical trial (NCT01894815). With this dataset, individualized neuroimaging-derived computational models of tDCS current were generated for (1) classifying treatment response, (2) elucidating essential stimulation features associated with treatment response, and (3) computing a personalized dose of tDCS to maximize the likelihood of treatment response in MDD.

**Results:**

In the ELECT trial, tDCS was superior to placebo (3.2 points [95% CI, 0.7 to 5.5; *P* = 0.01]). Our algorithm achieved over 90% overall accuracy in classifying treatment responders from the active-tDCS group (AUC = 0.90, F1 = 0.92, MCC = 0.79). Computed precision doses also achieved an average response likelihood of 99.981% and decreased dosing variability by 91.9%.

**Conclusion:**

These findings support our previously developed precision-dosing method for a new application in psychiatry by optimizing the statistical likelihood of tDCS treatment response in MDD.

**Supplementary Information:**

The online version contains supplementary material available at 10.1186/s42234-024-00157-2.

## Introduction

Major Depressive Disorder (MDD) is a complex and multifaceted mental health condition that affects hundreds of millions of individuals worldwide (Otte et al. 2016). MDD has a substantial impact on an individual’s daily functioning and quality of life, leading to difficulties in personal relationships, work productivity, and social activities (Bromet et al. 2011; James et al. 2018). Depressive disorders are the third largest cause of disability worldwide (James et al. 2018), and the economic burden associated with this condition is substantial (James et al. 2018; Greenberg et al. 2021). In 2018, the annual cost of MDD in the United States was estimated to be $326.2 billion, which is a nearly 40% increase from 2010 (Greenberg et al. 2021).

Despite the availability of different treatment options, such as medication and psychotherapy, nearly 31% of patients do not achieve remission (Gibson-Smith et al. 2015), and the long-term outcomes are often unsatisfactory (Nemeroff 2007). Moreover, many of these treatments have non-trivial side effects, including sexual dysfunction, insomnia, weight gain, etc. (Trivedi et al. 2006; Nemeroff 2007). In some cases, these side effects can lead patients to turn away from pursuing or continuing treatment. Due to the high prevalence and substantial burden of MDD, there is a growing need for alternative or complementary interventions that can provide effective and long-lasting relief for individuals with treatment-resistant depression (Trivedi et al. 2006; Nemeroff 2007; Warden et al. 2007).

One such treatment is transcranial direct current stimulation (tDCS), a non-invasive brain stimulation technique that involves the application of a weak electrical current over the scalp to modulate cortical excitability (Albizu et al. 2019). tDCS alters the sub-threshold membrane potential of neurons (Albizu et al. 2019) and increases regional blood flow while modulating local neurotransmitter concentrations during stimulation (Radman et al. 2009a; Fritsch et al. 2010; Reato et al. 2013; Podda et al. 2016; Kronberg et al. 2017; Antonenko et al. 2019; Alvarez-Alvarado et al. 2021). *In vivo* and *in vitro* studies have shown that the weak electric field induced by tDCS can modulate cortical excitability (Esmaeilpour et al. 2018), as well as alter synaptic plasticity (Podda et al. 2016; Kronberg et al. 2017, 2020). tDCS has shown promising results for treating various neuropsychiatric conditions (Szymkowicz et al. 2016; Clancy et al. 2018; Indahlastari et al. 2021; Kim et al. 2022), including MDD (Knotkova et al. 2012; Brunoni et al. 2013, 2017). Several randomized controlled trials for MDD have achieved varying results by targeting the hypoactive dorsolateral prefrontal cortex (DLPFC) with tDCS (Fregni et al. 2006; Bares et al. 2008; Boggio et al. 2008; Murphy et al. 2009; Nitsche et al. 2009; DMMsF et al. 2012; Kalu et al. 2012; Palm et al. 2012; Brunoni et al. 2013, 2017). Some studies have reported significant improvements in depressive symptoms compared to sham stimulation (Fregni et al. 2006; Bares et al. 2008; Boggio et al. 2008; Murphy et al. 2009; Nitsche et al. 2009; Kalu et al. 2012; Brunoni et al. 2013, 2017), while others have failed to demonstrate significant effects (DMMsF et al. 2012; Palm et al. 2012).

This may be due to the “one-size-fits-all” or fixed dosing approach adopted by most tDCS trials. Specifically, all participants are assigned to receive the same stimulation parameters (*e.g.,* F3/F4 at 2 mA) without regard to each individual’s unique anatomy. Thus, the level of variability in trial outcomes could be (at least partially) attributed to individual differences in head anatomy. For example, individual MRI-derived computational models of tDCS-induced current are linearly affected by anatomical attributes such as cortical atrophy (Indahlastari et al. 2020a), leukoaraiosis (Indahlastari et al. 2020b), skull thickness (Opitz et al. 2015), adiposity (Truong et al. 2013), etc. While these anatomical factors represent just one dimension of inter-individual variability, anatomical factors have the greatest effect on the distribution and targeting of tDCS, affecting individual response to tDCS (Bulubas et al. 2019; Albizu et al. 2020; Suen et al. 2020; Nandi et al. 2022). Although every brain possesses unique characteristics, variations in brain structure are also particularly evident in the presence of pathology, like depression (Kanner 2004; Palazidou 2012). However, the majority of research has investigated the impact of anatomical differences on tDCS response in healthy populations. Thus, there is a pressing need to investigate the predictive value of pathological differences in anatomy on treatment outcomes in tDCS. A method to account for individual anatomical differences in an MDD population may provide a unique pathway toward a precision medicine approach of tDCS for maximal therapeutic outcomes.

Considering inherent dosing variability across individuals, algorithms of machine learning (ML) can be applied to patient-specific, neuroimaging-derived computational models of electric current to address the heterogeneity of response to tDCS (Albizu et al. 2020, 2023; Kambeitz et al. 2020; Kim et al. 2022). While other studies have utilized machine learning to classify treatment response (Albizu et al. 2020, 2023; Kambeitz et al. 2020; Kim et al. 2022), only our previous results have leveraged this approach to optimize tDCS dosing (Albizu et al. 2020, 2023). Thus, the current study replicates our previous results in a new, clinical and affective domain by applying a machine learning approach to 1) classify individual response to tDCS application, 2) investigate the features of the electric field that are the best classifiers of treatment response, and 3) optimize tDCS parameters to maximize the likelihood of treatment response. Potential findings of this study may show promise in refining stimulation dosing strategy by accounting for individual anatomical differences. Precise targeting of brain regions in need of stimulation may potentially improve outcomes for patients with MDD.

## Methods

**Fig. 1:**
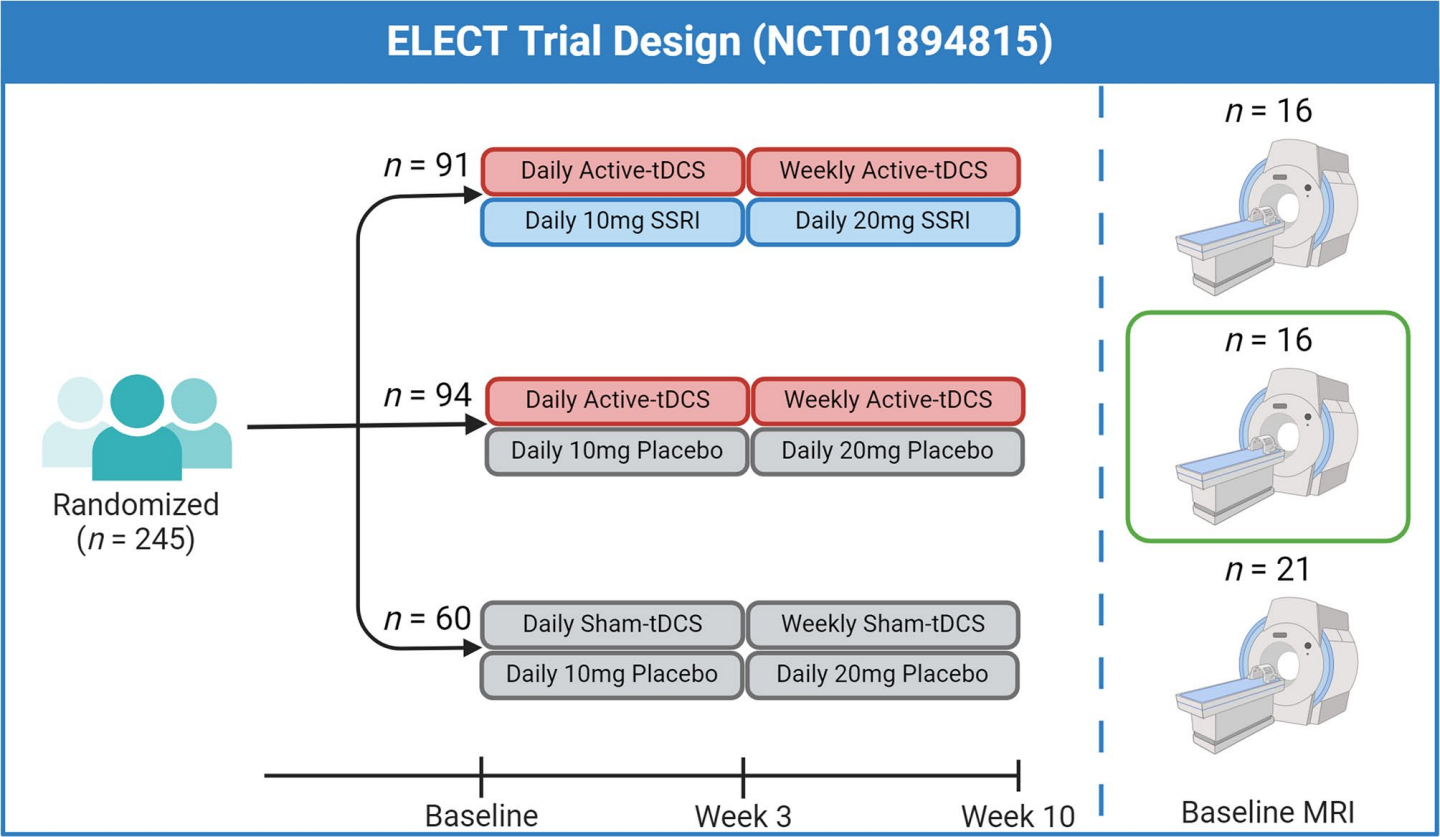
A schematic of the ELECT clinical trial design. The current study performed secondary analysis of the active-tDCS + placebo group with baseline MRIs (*n* = 16; green square). Created with Biorender.com

Structural imaging and behavioral data were sourced from a phase-III, randomized, non-inferiority, triple-arm, placebo-controlled study (NCT01894815; Fig. 1). In the trial, 245 MDD patients were recruited to one of three groups: active-tDCS with placebo pill, sham-tDCS with escitalopram, and sham-tDCS with placebo pill (Brunoni et al. 2015).

### Participants

16 patients with Major Depressive Disorder (MDD) receiving active-tDCS stimulation were selected (in accordance with a previous study (Suen et al. 2020)) for further analysis by the current study [mean (sd) age = 42.8 (10.9), mean HDRS-17 = 21.6 (3.9), 9 F:7 M]. Eligible patients were diagnosed with MDD according to DSM-5 criteria and confirmed by psychiatrists using the Mini-International Neuropsychiatric Interview (MINI) (Amorim 2000). Eligible patients were between 18–75 years old, had a score of 17 or higher on the 17-item Hamilton Depression Rating Scale (HDRS-17), and had a low suicide risk as determined by the MINI. Patients with bipolar disorder, substance abuse or dependence, dementia, a personality disorder, brain injury, a current pregnancy, specific contraindications to tDCS, current or previous use of escitalopram, or previous or concurrent participation in other tDCS trials were excluded. Coexisting anxiety disorders (generalized anxiety disorder, specific phobia, panic disorder, or social anxiety disorder) did not result in exclusion. Before the trial, patients were free of antidepressants or completed a washout. Benzodiazepines were limited to a stable dose of 20 mg/day of diazepam equivalent. The trial was conducted at the University Hospital and Department and Institute of Psychiatry, University of São Paulo.

### tDCS protocol and application

Using the Omni-Lateral-Electrode system (Seibt et al. 2015), the anode and cathode electrodes (5 × 5 cm^2^ pads) were positioned over the left and right dorsolateral prefrontal cortices, respectively (165° rostral and 10 cm apart near F5/F6 of the 10–20 system) (Brunoni et al. 2015). Each session involved a 30-minute application of 2 mA of tDCS (Soterix Medical, tDCS-CT for clinical trials) for 10 weeks. The first 15 sessions occurred daily, except for weekends, while the remaining 7 sessions were held once per week for a total of 22 sessions conducted. Patients in both the active and sham tDCS groups were subjected to the same protocol. The devices were pre-programmed with a randomized code to deliver either active or sham stimulation, However, in the sham group, the current was automatically turned off after 30 seconds.

### Treatment outcomes

Trained psychiatrists and psychologists, who were unaware of the trial-group assignments, conducted all assessments. All assessors were trained using the structured interview guide for the HDRS-17 and only started participating in the study if the reliability was > 90% compared to the gold standard. Efficacy and safety were evaluated at screening, baseline, and at the end of weeks 3, 6, 8, and 10. Positive class labels (n = 10) were defined as > 50% reduction between the baseline and 10-week timepoint on the HDRS-17, the Montgomery–Åsberg Depression Rating Scale (MADRS), and Positive and Negative Affect Scale (PANAS), similar to a previous study (Suen et al. 2020). No significant safety concerns were identified during the study. To assess the integrity of trial-group blinding, patients were asked to guess which intervention they had received and to rate the confidence in their prediction. Participants were unable to guess tDCS assignment ($\:{\chi\:}^{2}\left(2\right)=2.6,\:p=0.27$).

### Imaging sequences and parameters

Structural T1-weighted MRI scans were obtained on a 3T MR system (Achieva, Philips Healthcare, Netherlands). The 3D Fast Field Echo sequence parameters included: repetition time (TR) = 7 ms; echo time (TE) = 3.2 ms; flip angle = 8°; field of view (FOV) = 240 × 240 mm; resolution = 1 × 1 mm; slice thickness = 1 mm; and 180 sagittal slices. MR acquisitions were conducted between 4 and 8 days before baseline and took place at the Department of Radiology (Hospital das Clínicas da Universidade de São Paulo, São Paulo) during the weekends.

### Computational model construction

**Fig. 2:**
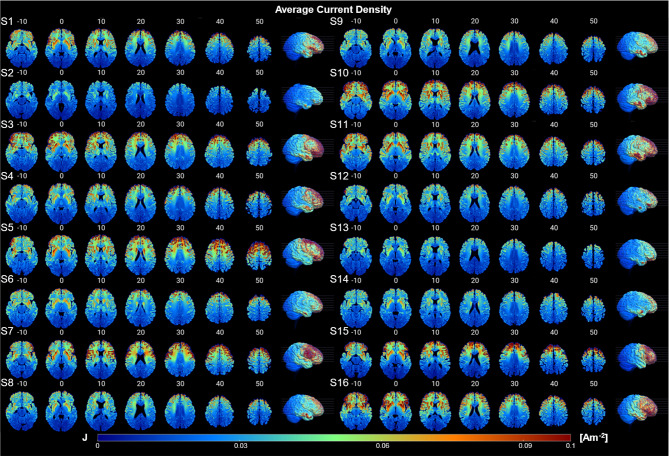
The spatial distribution of current density for all 16 participants

The individual T1-weighted images were converted into a 256 mm^3^ isometric field of view, 1 mm^3^ voxel size, and RAS orientation with the FreeSurfer v7.1.1 image analysis suite (http://surfer.nmr.mgh.harvard.edu/). Individual head volumes were segmented into six tissue types: white matter, gray matter, cerebrospinal fluid, bone, skin, and intracranial air with HEADRECO (Nielsen et al. 2018) – provided by SimNIBS v3.2.1 (https://simnibs.github.io/simnibs/build/html/index.html). Individual tissue types were assigned default isotropic conductivity values using the Realistic vOlumetric-Approach to Simulate Transcranial Electric Stimulation (ROAST; https://www.parralab.org/roast/) toolbox (Huang et al. 2019). A custom MATLAB 2022a (https://www.mathworks.com/) script was used to simulate electrodes according to the Omni-lateral Electrode System (Seibt et al. 2015). At these custom locations, 5 × 5 cm^2^ electrodes with + 2 mA/-2 mA boundary conditions were assigned as anode and cathode, respectively. A finite element solver, getDP, was used to compute voltage solutions to the Laplace equation. Additional MATLAB routines were used to compute current density from electric field and tissue conductivity, in accordance with Ohm’s law ($\:J=\sigma\:E$; Fig. 2). Further, to assess the importance of the current density magnitude, the current density direction, and the curren density vector (magnitude and direction) in classifying treatment response; we compare the classification performance of each data type (see supplemental materials for more information).

### Supervised machine learning

A Support Vector Machine (SVM) algorithm was used to classify the responders from non-responders, like our previous approach (Albizu et al. 2020). SVM searches for the optimal hyperplane that can separate two classes with maximal margin, under the assumption of independently and identically distributed (iid) data (Steinwart and Christmann 2008), which is met in this study. For the detailed machine learning methodology, please refer to the supplemental materials. To replicate our prior results, three data types: current density magnitude, direction, and vector were extracted to be utilized for classification (Albizu et al. 2020). For feature weight generation and deployment, a final model was trained on all 16 current density maps to derive overall classification weights. The feature weights at each voxel were separated by positive and negative weights that classify responders and non-responders, respectively. To identify the specific features that classify tDCS-related depressive symptom improvements, positive weights were normalized (i.e., $\:\sum\:{w}^{+}=1$) with the following equation to determine their percent contribution toward response classification:

1 $$\:{w}^{+}=\left\{\begin{array}{c}\frac{{w}_{i}\:}{\sum\:_{v:{w}_{v}>0}{w}_{v}},\:\:{w}_{i}>0\\\:0,\:\:\:\:\:\:\:\:\:\:\:\:\:\:\:\:\:\:\:\:\:\:\:\:\:\:\:\:{w}_{i}\le\:0\end{array}\right.$$

Regions of interest (ROIs) were defined using the Harvard-Oxford atlas [52], and regions were ranked based on the average weight of each voxel in that region, $\:{w}_{ROI}^{+}$: $\:\frac{1}{\left|v\in\:ROI\right|}\sum\:_{v\in\:ROI}{w}_{v}^{+}$, where $\:ROI$ represents the set of voxels within a specified region of the atlas.

### Dose optimization

**Fig. 3:**
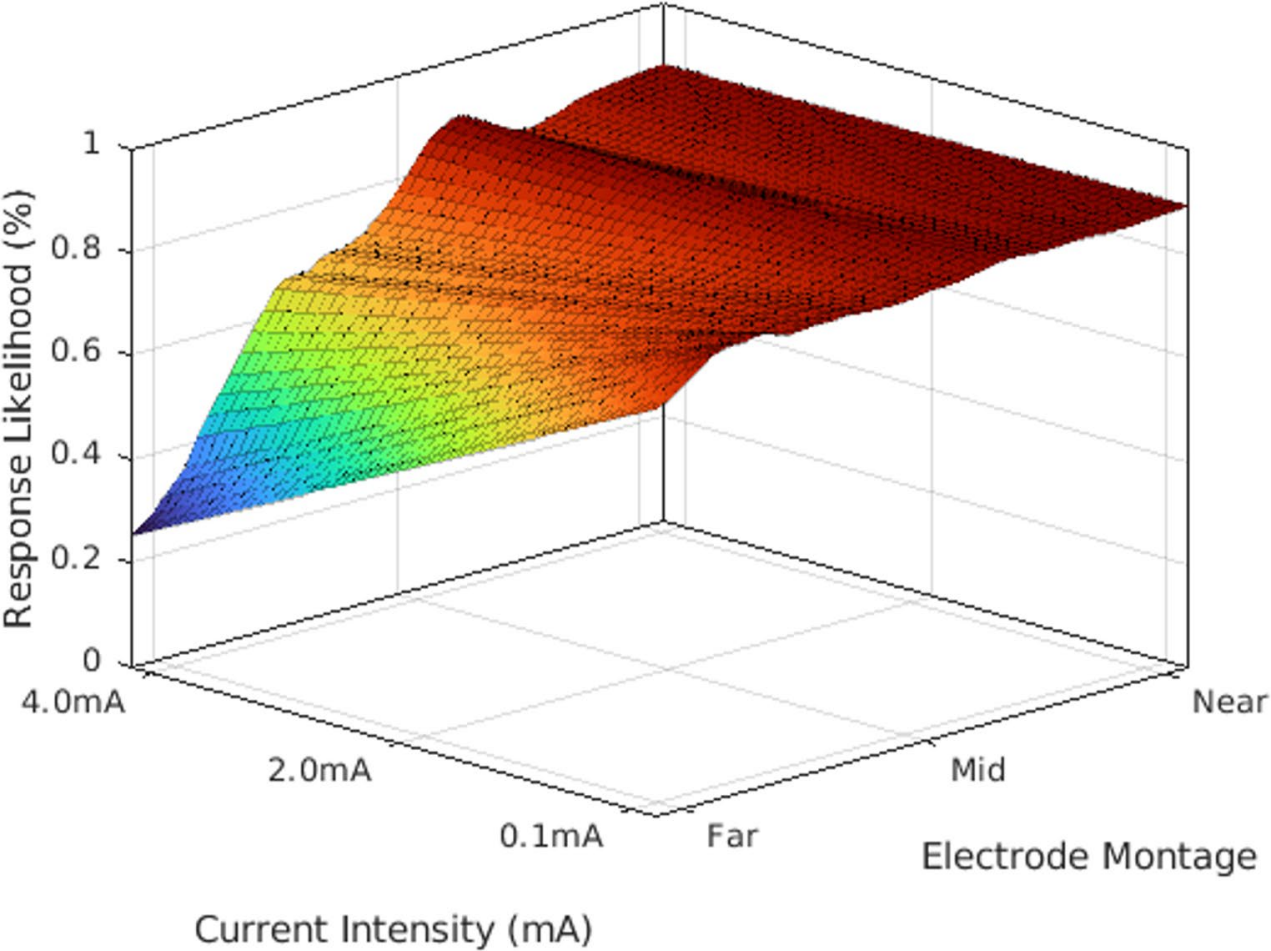
A representative optimization space for a single participant. A 5th-degree polynomial fit was applied to demonstrate the gradient of treatment response across multiple parameters. The x-axis represents the applied current intensity in milliamps. The y-axis represents the electrode montage (i.e., the bipolar electrode configuration) by distance from the originally applied montage (e.g., F5/F6). The z-axis represents the statistical likelihood of treatment response for a single participant given the specific combination of parameters

Learned weights of the SVM model were used in a modified, weighted Gaussian mixture model (GMM) to generate a precision model that accounts for inter-personal variation based on the current distribution of responders. The empirical responder mean, empirical responder variance, and SVM feature weights were used to estimate each Gaussian model. The likelihood (ℓ) of a new subject belonging to the responders’ current distribution (i.e., response likelihood) was calculated using:

2 $$\:\mathcal{l}\left(x\:|\:{w}^{+},\:\mu\:,\:\sigma\:\right)={e}^{-\sum\:_{v=1}^{V}\frac{{w}_{v}^{+}{\left({x}_{v}-{\mu\:}_{v}\right)}^{2}}{{\sigma\:}_{v}^{2}+1}}$$

where $\:{w}_{v}^{+}$ is the SVM response classification weight (s.t., $\:\sum\:{w}^{+}=1$), $\:{\mu\:}_{v}$ is the empirical responder mean, and $\:{\sigma\:}_{v}^{2}\:$is the empirical responder variance for the $\:{v}^{th}$ of $\:V$ features. The likelihood estimate was used as the objective function to optimize tDCS parameters (i.e., electrode placement and injected current intensity; see Fig. 3) for each individual’s unique head anatomy. In total, electrode positions were optimized from 71 locations from the 10–10 system (71 × 70 = 4,970 potential electrode pairs). Further, the injected current intensity was simulated in 0.1 mA increments up to 4 mA for a total of 40 possible input current levels. Thus, the overall tDCS optimization space included 198,800 potential tDCS doses per person. Normalized mutual information, feature-wise regression, and feature-wise dot product were used as metrics to evaluate the performance of optimization by comparing the similarity of the optimization results with the average treatment responder. For visualization of optimization performance, principal component analysis (PCA) was also utilized to project the expansive feature space and Gaussian model into two dimensions (i.e., the first two principal components). As an additional metric of optimization performance, current density volumes of optimized doses for non-responders were passed back through the original SVM model to assess reclassification of non-responders as responders following optimization.

### Statistical analyses

The Statistics and Machine Learning Toolbox in MATLAB 2022a was used to carry out statistical analyses. Hedges’ g was computed to define effect sizes of mean differences, corrected for small sample bias. Since all sixteen participants in our study were individuals with no familial relationship, and each participant’s data was collected under the same condition, these data points met the statistical assumptions of iid data.

## Results

The parent ELECT clinical trial of the current study primarily found a significant decrease in the HDRS-17 depression scores in both active-tDCS groups compared to sham-tDCS with placebo pill (tDCS with escitalopram: 5.5 points [95% CI, 3.1 to 7.8; *P* < 0.001; Response rate: 47%], tDCS with placebo pill: 3.2 points [95% CI, 0.7 to 5.5; *P* = 0.01; Response rate: 41%]) in the intent-to-treat analysis.

### Machine learning classification of tDCS intervention efficacy

**Fig. 4:**
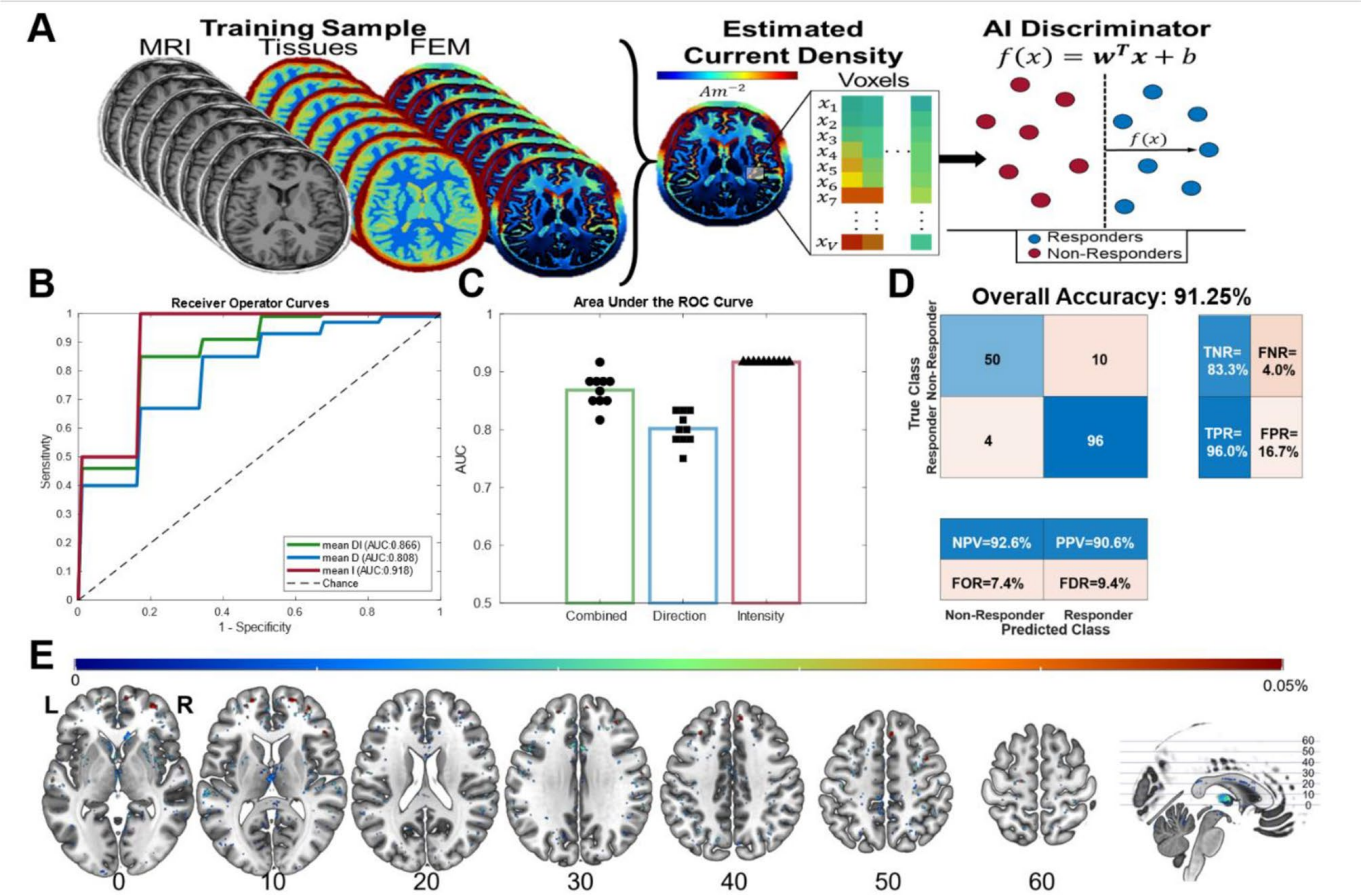
(**A**) A schematic diagram of the treatment response classification workflow. (**B**) The mean raw and fitted ROC curve of each data type across ten iterations. (**C**) The AUC of the ROC curve for each of the data types across ten iterations. (**D**) an aggregate confusion matrix across ten iterations for the best performing model. (**E**) Discrimination maps of regions that classify treatment response with the percent contribution of each voxel to the SVM decision function, superimposed onto the MNI152 Template

In this study, we used a Support Vector Machine (SVM) learning algorithm to distinguish tDCS responders from non-responders based on modeled current density magnitudes, directions, and vectors (a combination of magnitudes and directions). Here, the SVM model accurately distinguished tDCS responders from non-responders using current density magnitudes, achieving an overall accuracy of 91.25% [95% CI 88.54% – 93.96%] with an area under the curve (AUC) of 0.90, an F1 score of 0.93, and a Matthews correlation coefficient (MCC) of 0.82 (see Fig. 4). We also found that current density magnitudes outperformed current density vectors and current density directions in classifying treatment response ($\:F\left[\text{2,29}\right]=65.08,p<0.001$). Specifically, the accuracy of the model based on current density vector was 85%, with an AUC of 0.89, an F1 score of 0.88, and an MCC of 0.69. The accuracy of the model based on current density direction was 81%, with an AUC of 0.85, an F1 score of 0.86, and an MCC of 0.59 (see Table 1 for full results). These results indicate that current density magnitudes are a reliable classifier of tDCS treatment response in MDD.

**Table 1:** A summary of the ablation experiment with SVM model performance per data type

	Intensity	Direction	Combined
Accuracy	**0.913**	0.688	0.788
AUROC	**0.918**	0.808	0.866
F1 Score	**0.932**	0.795	0.835
MCC	**0.812**	0.301	0.539
Sensitivity	0.960	**0.970**	0.860
Specificity	**0.833**	0.217	0.667
FPR	**0.167**	0.783	0.333
FNR	0.040	**0.030**	0.140

AUROC – Area Under the Receiver Operating Characteristic Curve, MCC – Matthews Correlation Coefficient, FPR – False Positive Rate, FNR – False Negative Rate

### Electric Field characteristics to Classify Treatment Response

**Fig. 5:**
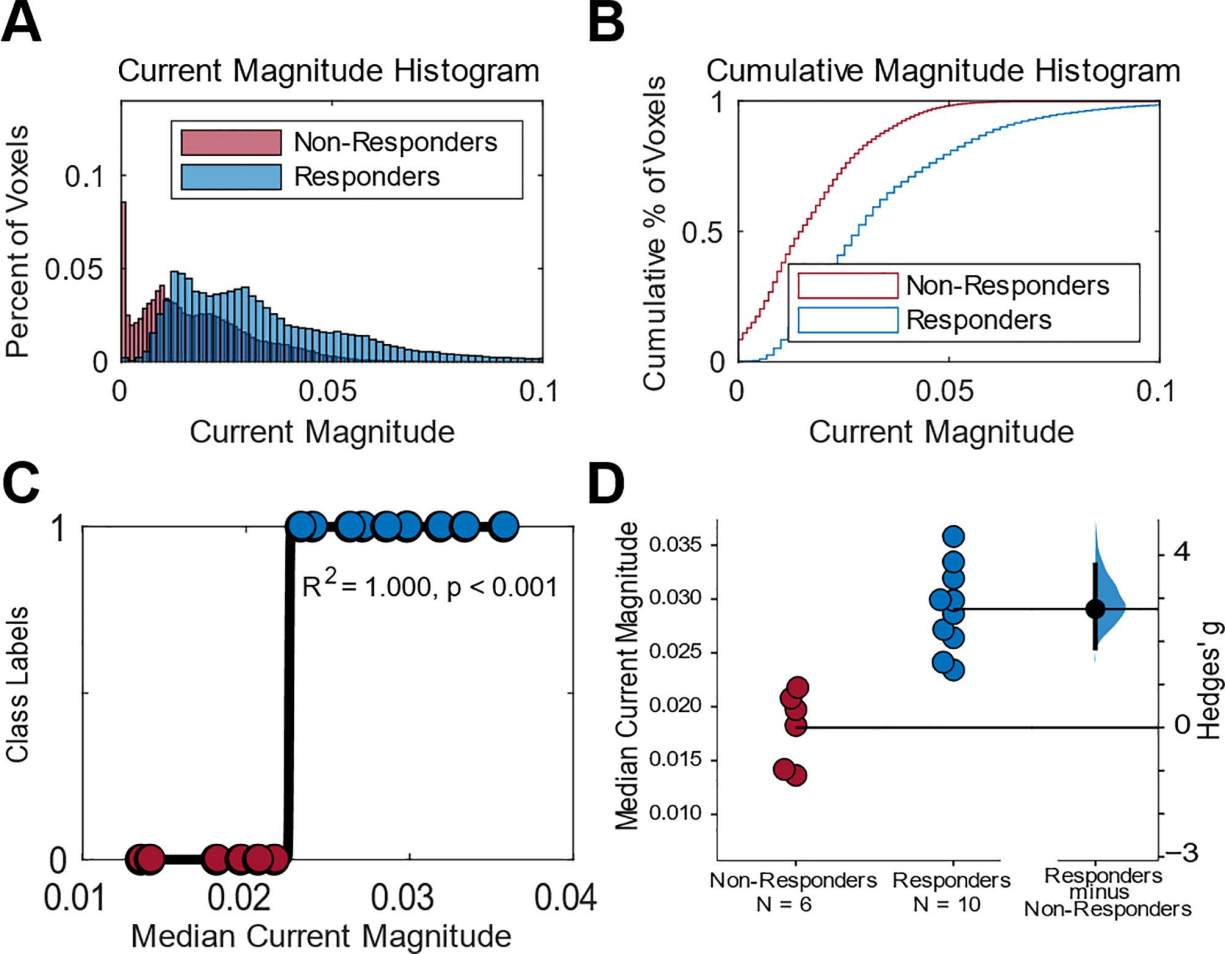
Plots to demonstrate the current density characteristics within regions predictive of tDCS responders. (**A**) Histogram of current magnitude (bin width of 0.0013 Am^-2^), with the y-axis representing the number of observations in each bin divided by the total number of observations, where the sum of all bar heights is equal to 1. (**B**) Cumulative histogram of current magnitude with the height of each step equal to the cumulative number of observations in the bin over the total number of observations in each bin and all previous bins where the height of the last bar is equal to 1. (**C**) Logistic regression of class labels vs. median current magnitude. Each dot represents a single participants median current magnitude. (**D**) The Hedges’ g between responders and non-responders is shown in a Gardner-Altman estimation plot. Each dot represents a single participants median current magnitude. The mean difference is plotted on a floating axis as a bootstrap sampling distribution. The mean difference is depicted as a dot; the 95% confidence interval is indicated by the ends of the vertical error bar

Furthermore, we examined the brain voxels that classify tDCS responders (i.e., the feature weights learned during training, $\:{w}^{+}$ from Eq. 1). As shown in Fig. 5, the median current magnitude was significantly higher in responders than in non-responders within these brain regions ($\:r\:=\:0.999,\:p\:<0.001$, see Fig. 5C), indicating a strong positive relationship between current density magnitude and treatment response. To quantify the difference in current density magnitudes between responders and non-responders, we calculated the effect size using Hedges’ $\:g$. The effect size was 2.75, with a 95% confidence interval between 2.08 and 4.46 ($\:F\left[\text{1,15}\right]\:=\:31.8,\:p\:<\:0.001$; see Fig. 5D), indicating a large and reliable difference between the two groups. These results suggest that current magnitude is a robust classifier of tDCS treatment response, and that higher current magnitudes within essential brain regions may be associated with a greater likelihood of response.

### Regional contributions toward classification of tDCS response

**Fig. 6:**
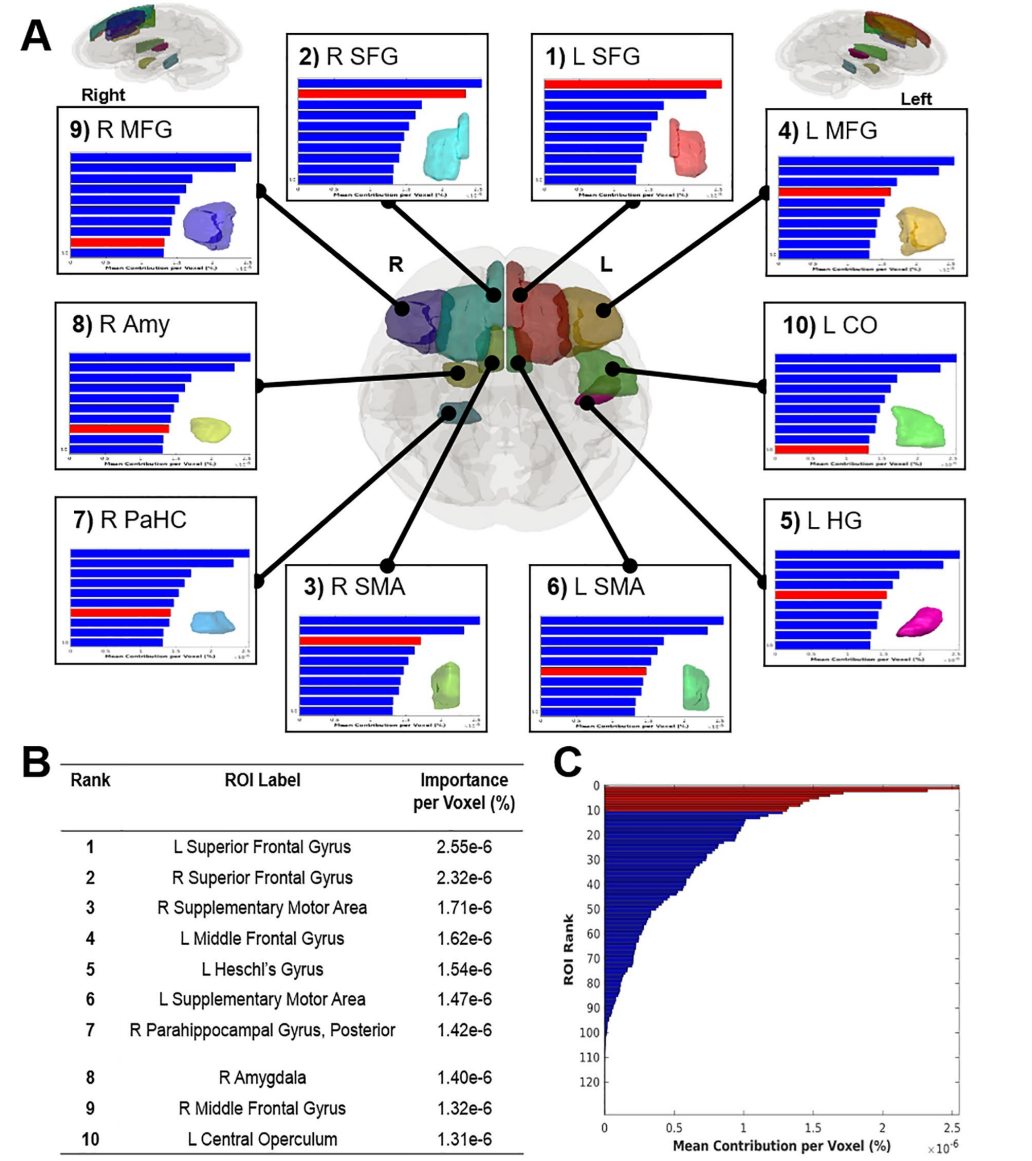
(**A**) Visualization of ROIs from the Harvard-Oxford atlas ranked based on their contribution toward predictions of treatment response. (**B**) Rank, label, and mean percent contribution per voxel of each ROI. (**C**) A bar graph to represent the average percent contribution per voxel within each ROI of the Harvard-Oxford atlas. Within the bar graph, the top ten ROIs are highlighted in red

To visualize the regions of the brain contributing to treatment response, Fig. 6 illustrates the top ten regions of interest (ROIs) from the Harvard-Oxford atlas, based on the average normalized weight per voxel within each ROI. The distribution of percent contribution across Harvard-Oxford ROIs is shown in Fig. 6B and C. The top ranked ROIs that classified depressive symptom improvements were largely located in the prefrontal and medial temporal lobes, as shown in Fig. 6A. These ROIs were labeled as the: (1) Left Superior Frontal Gyrus, (2) Right Superior Frontal Gyrus, (3) Right Supplemental Motor Area, (4) Left Middle Frontal Gyrus, (5) Left Heschl’s Gyrus, (6) Left Supplementary Motor Area, (7) Right Posterior Parahippocampal Gyrus, (8) Right Amygdala, (9) Right Middle Frontal Gyrus, (10) Left Central Operculum (Fig. 6). Overall, these brain regions have been implicated in emotion regulation, cognitive control, and sensory processing, all of which are disrupted in depression.

### Dose optimization performance

**Fig. 7:**
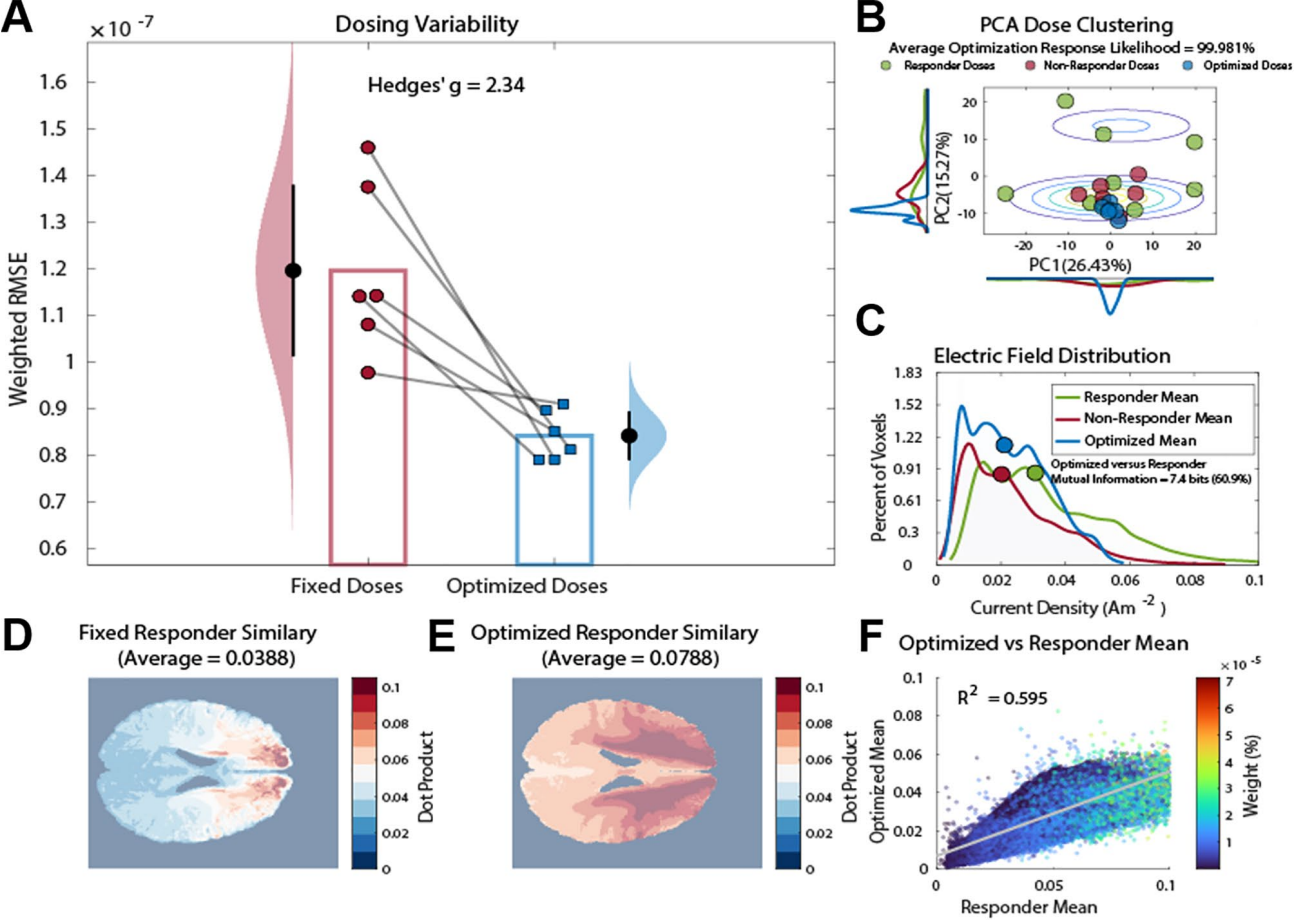
Conventional fixed dosing compared to optimized dosing. (**A**) Dosing variability measured by root mean squared error (RMSE) compared to the responder current density within the brain for fixed versus optimized doses, respectively. Black dots represent the mean values. Error bars represent ± 1 SD from the mean values. Histograms represent the normal distribution of the sample. (**B**) Response likelihood of responder (green), non-responder (red), and optimized (blue) models. Contour lines represent a 3D Gaussian distribution of the first and second principal components (i.e., PC1 and PC2) for responders. Histograms represent the smoothed distribution of PC1 and PC2 for estimated current density. (**C**) Estimated current density reaching the brain for responder (green), non-responder (red), and optimized (blue) dosing. Dots represent the median of each distribution. (**D**) 3D dot product of the mean current density vector for the fixed versus responder mean. (**E**) 3D dot product of current density vectors for average optimized dose versus average responder dose. (**F**) Scatter plot of the voxel-wise mean current density of optimized doses versus the voxel-wise mean current density of tDCS responders

To correct the differences in current density between responders and non-responders, a GMM (i.e., Eq. 2) was used to compute and optimize the likelihood of treatment response given the estimate current density within the brain. An exhaustive search of the tDCS optimization space was used to identify the global optimum dosing parameters for non-responders to match the current profile of treatment responders. Following optimization, original non-responders were significantly closer to the responder current density mean by 2.34 pooled standard deviations compared to pre-optimization fixed dosing of non-responders (see Fig. 7A, $\:F\left[\text{1,11}\right]=20.59,\:p=0.001,\:g=2.34,\:95\%\:CI=1.57-4.73$). GMM dose optimization also achieved an average response likelihood of 99.981% (see Fig. 7B), indicating that the optimized doses were highly likely to lead to a positive treatment response. Furthermore, the optimized doses shared 60.9% (7.4 bits) of normalized mutual information with the mean current distribution for responders, indicating that the optimized doses were similar to those given to responders (see Fig. 7C). Regression of the mean optimized current density vector demonstrated strong feature-wise coherence (see Fig. 7F, $\:{R}^{2}=0.595,\:p<0.001$), which suggest that the optimized doses exhibited patterns of electrical current indicative of treatment response. Additionally, the GMM optimization decreased dosing variability by 91.9%, suggesting that the optimized doses were more consistent than non-optimized fixed doses. Furthermore, the optimized doses exhibited 203.4% greater average feature-wise dot product with the current density vector of responders, indicating that the optimized doses were more similar to those given to responders than conventional non-responder fixed doses (see Fig. 7D-E). Finally, the optimized doses of the non-responder group were passed back through the original SVM discriminator and 100% of these optimized doses were predicted to produce treatment responders, indicating a high level of classification accuracy and effectiveness of the optimization strategy. Overall, these results demonstrate that dose optimization maximized the likelihood of treatment response by consistently matching the current density distribution of treatment responders throughout the brain.

## Discussion

The results of this study suggest that machine learning algorithms applied to computational models of tDCS can effectively classify and optimize treatment response in individuals with major depression. Specifically, the current study applied a machine learning approach to (1) classify individual response to tDCS application, (2) investigate the features of the electric field that are the best classifiers of treatment response, and (3) optimize tDCS parameters to maximize the likelihood of treatment response. Our findings highlight the potential for machine learning algorithms to identify classification markers of response and prospectively adjust dosing parameters for optimal treatment outcomes.

### Response classification and current characteristics

The classifcation capabilities of this approach may be particularly useful in identifying individuals who are likely to benefit from tDCS and in developing personalized treatment approaches. Furthermore, our findings suggest that the magnitude of the electric field is the most effective classifier of treatment response, outperforming current density vector and direction. Specifically, responders were found to have greater current density magnitudes within the voxels that discriminate tDCS responders from non-responders. This finding suggests that the overall magnitude of current delivered may be a critical factor in the therapeutic effects of tDCS. Other current modeling studies have observed similar dose-response relationships to further support this notion (Amorim 2000; Seibt et al. 2015; Chauhan et al. 2018; Göksu et al. 2018; Antonenko et al. 2019; Suen et al. 2020). However, it is important to note that the direction of current may still play an important role in determining response. Both normal and tangential current have been demonstrated as essential components of current (Radman et al. 2009b; Rahman et al. 2013; Lafon et al. 2017; Rawji et al. 2018). Thus, future studies should integrate both current magnitude and directional components for a more comprehensive understanding of how tDCS modulates brain activity and improves treatment efficacy.

The use of machine learning algorithms in the context of tDCS treatment for major depression has several potential benefits. By identifying classification markers of response, machine learning algorithms may help to reduce reliance on fixed dosing or trial-and-error approaches currently favored in tDCS treatment, which can lead to suboptimal treatment outcomes and prolong the duration of treatment. Additionally, machine learning algorithms can be used to prospectively adjust dosing parameters to improve treatment outcomes, potentially leading to more efficient and effective treatment.

### Regions of importance

To that end, our study identified specific ROIs that were effective classifiers of response to tDCS. These ROIs were primarily located in the prefrontal and medial temporal lobes, with the bilateral superior frontal gyri, parahippocampal gyrus, and amygdala being within the top 10 best classification ROIs. The prefrontal cortex (PFC) is involved in regulating mood and emotion (Drevets 2007; Pizzagalli 2014), as well as cognitive functions such as decision-making, problem-solving, and attention. Studies have shown that individuals with MDD have decreased activity in the PFC (Grimm et al. 2008; Diener et al. 2012), particularly in the dorsolateral prefrontal cortex (DLPFC) and anterior cingulate cortex (ACC). The importance of frontal regions is also likely a result of those being the areas targeted by the stimulation. The amygdala, on the other hand, is a key brain region involved in processing emotions, particularly fear and anxiety. Research has suggested that individuals with MDD may have increased activity in the amygdala, leading to heightened emotional reactivity and sensitivity to negative stimuli (Whalen et al. 2002; Diener et al. 2012). Our findings of the best classification ROIs are consistent with previous research suggesting that prefrontal and medial temporal lobes play a critical role in the pathophysiology of depression and the mechanism of action of tDCS (Whalen et al. 2002; Goldstein et al. 2007; Grimm et al. 2008; Diener et al. 2012; Lindquist et al. 2017).

### Dose optimization

The use of GMM optimization to personalize dosing for individual patients was also effective in computationally improving treatment response. Our results showed that GMM optimization led to significantly increased statistical response likelihood and decreased dosing variability compared to conventional fixed dosing for non-responders. Additionally, after receiving the optimized dose using our GMM method, the non-responder group were passed back through the original SVM discriminator, and 100% of them were classified to produce the same level of current distribution as the responder group. These findings suggest that the optimized dosing approach may be a promising method for improving the efficacy of tDCS for the treatment of depression.

### Study limitations

Despite the promising findings of this study, several limitations should be noted. First, the sample size of this study was relatively small, which may limit the generalizability of our findings. Future studies with larger sample sizes are needed to validate our results and to determine the clinical applicability of our findings. Additionally, the current study used a retrospective design, which limits our ability to draw causal inferences about the relationship between tDCS parameters and treatment response. Prospective and controlled precision-dosing studies are needed to confirm the causal relationship between tDCS parameters and treatment response. Another limitation of our study is the use of computational models to estimate the electric field distribution. Although computational models are widely used in tDCS research, they are based on simplified assumptions (e.g., tissue segmentation, isotropic conductivity values, ideal electrode placement, etc.) and may not fully capture the complexity of the electrical properties of the brain. Utilizing neuronavigated electrode placement and modeling white matter anisotropy might improve these results in future studies. Furthermore, our current optimization approach primarily accounts for physical differences, such as anatomical variations, in a multidomain problem where functional and physiological factors may also play critical roles. Accounting for additional domains, such as combining structural MRI (sMRI) and functional MRI (fMRI) data, would likely improve the predictive accuracy and efficacy of individualized tDCS dosing. Finally, the need for MRI scans to perform personalized treatment planning could be cost prohibitive in a clinical setting; if the results of the study prove marginal, the cost of MRI would not justify this approach. Thus, our findings should be interpreted with caution and validated using other methods, such as in-vivo current density imaging and randomized control trials (Kasinadhuni et al. 2017; Göksu et al. 2018).

### Conclusion

In summary, the ability of the current approach to identify classification markers of tDCS response and adjust dosing parameters accordingly may enable clinicians to optimize treatment outcomes for individual patients. This approach may also help to address the issue of variability in treatment response across individuals, which is a common challenge in the treatment of depression. The optimized dosing approach achieved an average response likelihood of 99.981% and significantly decreased dosing variability by 91.9% compared to conventional non-responder fixed doses. Furthermore, our findings suggest that the magnitude of the electric field within the brain is an effective classifier of treatment response, demonstrating the importance of optimizing tDCS parameters to account for individual anatomical differences. Our findings highlight the potential for machine learning algorithms to improve the effectiveness of tDCS treatment for depression. However, further research is needed to validate our findings and to determine the clinical applications of this approach. Nonetheless, these findings have important implications for the development of personalized tDCS dosing regimens for the treatment of depression.

## Electronic supplementary material

Below is the link to the electronic supplementary material.

Supplementary Material 1

## Data Availability

The processed data of this study are available upon request from the corresponding author. The raw data are not publicly available due to potential identifying information that could compromise participant privacy. Source data are provided with the paper in a public repository (https://github.com/aa14av/BioElecMed_Albizu_2024/).
