# Supplementary material for: Machine learning-optimized non-invasive brain stimulation and treatment response classification for major depression
Source: Bioelectron Med. 2024 Oct 30;10:25. doi: 10.1186/s42234-024-00157-2 (PMC11524011; doi:10.1186/s42234-024-00157-2)
Supplement: Supplementary file 1 — Supplementary Material 1 [file 42234_2024_157_MOESM1_ESM.docx]

**Supplemental Materials**

**Machine Learning Methodology**

To classify the responders from non-responders, the Support Vector Machine (SVM) algorithm was used. SVM searches for the optimal hyperplane that can separate two classes with maximal margin, under the assumption of independently and identically distributed (iid) data (54), which is met in this study. For the machine-learning methodology, please refer to the supplemental materials. Specifically, LIBSVM (55) was used to optimize the objective function:

|  | $\min_{w,b} \frac{1}{2}w^{T}w+C\sum_{n=1}^{N} \max\left( 1-y_{n}\left( w^{T}x_{n}+b \right),0 \right)$ | (1) |
| --- | --- | --- |

where $C\geq0$ is a penalty parameter on the training error. $y_{n}$ and $x_{n}$ are the ground truth label and feature vector for the $n^{th}$ of $N$ total observations, respectively. The SVM model was trained to optimize parameters $w$ and $b$, which represent the weight and bias, respectively.

Model performance was evaluated across 10 permutations of two-level nested stratified cross-validation (CV) (56). The dataset was split into $K = 8$ folds for the outer CV loop. For each permutation, we performed the following steps:

1. Split the dataset into $K$ folds: $D = \{D_{1}, D_{2}, ..., D_{K}\}$.
2. For each fold $k$ in $D$:

A. Let $D_{k}$ be the $k$^th^ fold in the current permutation.

B. Let $D_{train}= \{D_{i} | i \neq k\}$ be the remaining folds used for training data.

C. Perform a CV loop on $D_{train}$ to find the optimal value of hyperparameter, $c$:

i. Let $C = \{c_{1}, c_{2}, ..., c_{m}\}$ be the set of $m$ possible values of $c$:

ii. For each $c$ in $C$, split $D_{train}$ into $K-1$ folds: $G = \{G_{1}, G_{2},\ldots,G_{K-1}\}$

iii. For each fold $j$ in $G$, let $G_{val}= \{G_{i} | i \neq j\}$ be the remaining folds used for validation.

iv. Train the model on $G_{val}$ using $c$.

v. Calculate the model performance $p_{j}^{c}$ on the validation set, $G_{j}$.

vi. Compute the average model performance using $c$ across all folds in G. $\bar{p}^{c}=\frac{1}{K-1}\sum_{j=1}^{K-1} p_{j}^{c}$

vii. Choose the value $c_{best}$ in $C$ that gives the best model performance $\bar{p}^{c}$.

D. Train the model on $D_{train}$ using the optimal value $c_{best}$

E. Use the trained model to predict labels for all participants in $D_{k}$.

F. Calculate performance metrics $P_{k}$ by comparing the predicted labels to the ground truth labels.

3. Calculate the average performance metrics $\bar{P}$ across all folds $D_{k}, k=1,2,\ldots, K$.

The above steps were repeated for each permutation, and performance metrics were averaged across all permutations. By using two-level nested stratified CV, we ensured that the validation and test sets were balanced and representative of the overall dataset, while also optimizing the hyperparameter $C$ in a robust and unbiased manner.

**REFERENCES**

54. Diener, C. *et al.* A meta-analysis of neurofunctional imaging studies of emotion and cognition in major depression. *Neuroimage* **61**, 677–685 (2012).

55. Whalen, P. J., Shin, L. M., Somerville, L. H., McLean, A. A. & Kim, H. Functional neuroimaging studies of the amygdala in depression. *Semin Clin Neuropsychiatry* **7**, 234–242 (2002).

56. Lindquist, M. A. *et al.* Group-regularized individual prediction: theory and application to pain. *Neuroimage* (2017) doi:10.1016/j.neuroimage.2015.10.074.

Please also refer the following references for additional methodological information:

Albizu, A. *et al.* Machine learning and individual variability in electric field characteristics predict tDCS treatment response. *Brain Stimul* (2020) doi:10.1016/j.brs.2020.10.001.

Albizu, A. *et al.* Machine-learning defined precision tDCS for improving cognitive function. (2023) doi:10.1016/j.brs.2023.05.020.
